# The Mechanical Characteristics of High-Strength Self-Compacting Concrete with Toughening Materials Based on Digital Image Correlation Technology

**DOI:** 10.3390/ma16041695

**Published:** 2023-02-17

**Authors:** Zhiqing Cheng, Hong Zhao, Guangcheng Long, Kai Yang, Mengting Chen, Zhi Wu

**Affiliations:** 1 School of Civil Engineering, Central South University, Changsha 410075, China; 2 Yunnan Traffic Science Research Institute Co., Ltd., Kunming 650011, China; 3 School of Materials Science and Engineering, Hunan Institute of Technology, Hengyang 421002, China

**Keywords:** high-strength self-compacting concrete, toughening materials, strength, crack, digital image correlation (DIC)

## Abstract

Brittle fracture is a typical mechanical characteristic of high-strength self-compacting concrete, and the research on its toughening modification remains the highlight in the engineering field. To understand the effect of toughening materials (including polymer latex powders, rubber particles, and polyethylene fibers) on the mechanical behavior of C80 high-strength self-compacting concrete under static loading, the failure mode, mechanical strength, strain field, and crack opening displacement (COD) of prepared high-strength self-compacting concrete under compressive, splitting, and flexural loads were studied based on digital image technology (DIC). The corresponding mechanism is also discussed. The results show that the hybrid of polymer latex powders, rubber particles, and polyethylene fibers can increase the crack path and inhibit the development of macrocracks in concrete, thus turning the fracture behavior of concrete from brittle to ductile. The addition of toughening materials reduced the compressive and flexural strengths of high-strength self-compacting concrete, but it increased the splitting strength. DIC showed that the incorporation of toughening materials promoted the redistribution of strain and reduced the degree of strain concentration in high-strength self-compacting concrete. The evolution of COD in high-strength self-compacting concrete can be divided into two stages, including the linear growth stage and the plastic yield stage. The linear growth stage can be extended by incorporating toughening materials. The COD and energy absorption capacity of concrete were enhanced with the addition of toughening materials, and the best enhancement was observed with the hybrid of polymer latex powders, rubber particles, and polyethylene fibers. Overall, this research provides a reference for exploring effective technical measures to improve the toughness of high-strength self-compacting concrete.

## 1. Introduction

Concrete is currently the most widely used engineering material. It has the advantages of easy access to raw materials and excellent mechanical properties, but it also exhibits the disadvantages of low tensile strength, high brittleness, and easy cracking. In the field of transportation engineering, the problem of durability in concrete structures has been the focus of attention due to the high brittleness and easy cracking of concrete [1,2,3]. In order to reduce the brittleness and cracking of concrete, a variety of toughening materials, such as fibers, polymers, and rubbers, have been used to prepare concrete. From the aspect of the particle sizes of materials, polyethylene fiber and rubber particles were macro toughening materials and polymer latex powder was a kind of micro toughening material. C.X. Qian et al. [4] studied the influence of the fiber size and fiber content on the mechanical properties of concrete and found that the different sizes of steel fibers contributed to different impacts on the mechanical properties of concrete. M.C. Nataraja et al. [5] and H. Zhou et al. [6] investigated the mechanical properties of fiber-reinforced concrete and found that the toughness and crack resistance of concrete had been improved. Y.P. Liu et al. [7] used a liquid epoxy resin–asphalt composite emulsion to pretreat aggregates and then prepared a polymer-modified concrete. The results showed that increasing the polymer film thickness on the aggregate surface could significantly increase the energy consumption of concrete during the fracture process. K.L Ma et al. [8] developed a self-compacting concrete with excellent workability by adding rubber particles, and the developed concrete exhibited a higher capability of resistance to chloride ion permeability compared to concrete without rubber particles. The above investigations have shown that incorporating the above-mentioned toughening materials can improve the toughness, crack resistance, and durability of concrete to varying degrees.

As we all know, concrete is a multi-phase composite material, and the incorporation of a single toughening material can only improve the performance of concrete in one aspect. If the toughness of concrete is to be enhanced comprehensively, the use of multi-scale toughness materials needs to be considered. For instance, some scholars [9,10,11] have studied hybrid fibers incorporated into concrete for strengthening and toughening, and some other scholars [12,13] have considered mixing fibers and polymer latex powders into concrete for performance improvement. There have also been investigations into using a hybrid of fibers and rubber particles in concrete [14,15] to enhance the mechanical properties and durability. The above studies utilized different toughening materials with different advantages for the comprehensive synergistic toughening of concrete. However, the effects of hybrid toughening materials (i.e., three toughening materials) on the properties of concrete has rarely been studied.

Moreover, concrete for transportation roads is also undergoing continuous technological updating with the rapid development of the transportation industry. As an example, high-strength self-compacting concrete is easy to construct and exhibits excellent performance. Thus, it is increasingly used in civil engineering [16,17]. However, the disadvantage of brittleness is prominent in high-strength self-compacting concrete [18,19]. The toughening of high-strength self-compacting concrete is imminent. In addition, digital image correlation (DIC) technology has been widely used in the observation and quantitative assessment of crack expansion and strain in concrete. DIC provides the advantages of full-field, non-contact measurements and high accuracy [20,21], and the brittle behavior of concrete is closely related to the evolution of cracks and deformations under load. Thus, DIC is suitable for the characterization of crack expansion in high-strength self-compacting concrete. According to the above description, scholars have clearly confirmed the effectiveness of toughening materials in improving the performance of concrete. However, there is still a need for further study of the mechanical performance in high-strength self-compacting concrete reinforced with hybrid toughening materials.

Given the above, the aim of this research was to explore the effective way to enhance the toughness of high-strength self-compacting concrete and to investigate the toughening effect of hybrid toughening materials on the performance of high-strength self-compacting concrete. For this objective, a series of tests, including compressive, splitting, and three-point bending tests, were carried out on the high-strength self-compacting concrete containing macro and micro toughening materials. Accordingly, the mechanical behaviors and deformation characteristics of high-strength self-compacting concrete containing hybrid toughening materials were quantitatively evaluated and analyzed based on DIC technology. Overall, this work can provide new ideas and references for the preparation of high-toughness and high-strength concrete for transportation engineering.

## 2. Experimental Programs

### 2.1. Raw Materials and Mix Proportions

**Figure 1:**
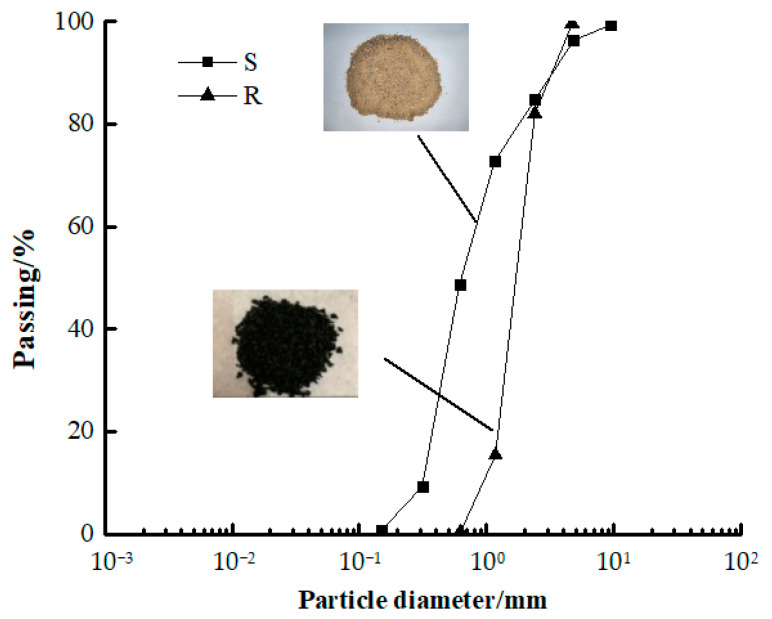
Particle size distributions.

In this experiment, Portland cement (PC, P·O 42.5), fly ash (FA), and silica fume (SF) were used as the binder materials, and the chemical composition and physical properties of the binder materials are shown in Table 1. River sand (S) was used as a fine aggregate with a fineness modulus of 2.62. Continuously graded crushed stone (G) was used as coarse aggregate with a particle size of 5–20 mm. A polycarboxylate-based superplasticizer (SP) was added as an additive with a water-reducing rate of around 33 %. Tap water (W) was used for mixing. Moreover, three toughening materials, including polyethylene fibers (F), rubber particles (R), and polymer latex powders (A), were used. In particular, polyethylene fibers (F) with diameters of 7–150 μm, lengths of 24 mm, an elastic modulus value of 100 GPa, and a tensile strength of 3000 MPa were used. Rubber particles (R) with particle sizes of 2.36–4 mm, an apparent density of 1090 kg/m^3^, and an elastic modulus value of 3.4 MPa were used. A polymer latex powder (A) with the particle size of 80 μm and an apparent density of 950 kg/m^3^ was used, and its main component was a copolymer of ethylene vinyl acetate. The mix proportions and fresh properties of the concrete are listed in Table 2. The particle size distributions of S and R are shown in Figure 1. 

### 2.2. Preparation and Curing of Specimens

The concrete strength grade of 80 MPa was designed in this work. The recommended dosage of toughening materials (R, A, and F) referred to the previous experimental research [8,22,23]. In particular, 10% of the volume of rubber particles was replaced by an equal volume of sand, and 10% of the volume of the polymer latex powder was replaced by an equal volume of binder materials (i.e., PC, FA, and SF). The volume content of polyethylene fiber was 0.3%. Moreover, the preparation process was as follows: First, all solid materials, including PC, FA, SF, S, G, A (if any), R (if any), and F (if any), were dry-mixed for 1 min. Then, the premixed solution, including W and SP, was slowly added, and mixing was continued for 2 min. After that, the workability (i.e., extensibility and T_500_) of the fresh mixture was tested, and the fresh mixture was poured into molds. Then, 100 mm × 100 mm × 100 mm specimens were formed for compressive and splitting strength tests, and 100 mm × 100 mm × 400 mm specimens were formed for a three-point bending test. Three specimens were prepared for each test. The specimens were stored at room temperature and covered with plastic films to prevent the evaporation of the free water in the mixtures. After 24 h, the specimens were demolded and then subjected to standard curing (20 ± 1 ℃ and >95 % RH) for 56 d.

### 2.3. Experimental Methods

#### 2.3.1. Mechanical Properties Test

**Figure 2:**
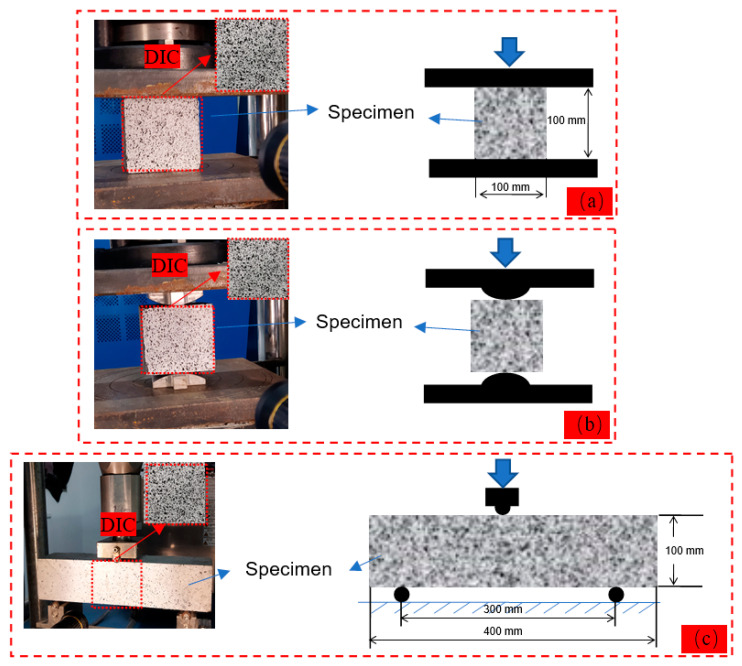
Test device of (**a**) compressive strength test, (**b**) splitting strength test, and (**c**) three-point bending test.

The compressive strength and splitting strength tests were stress-controlled, and the loading speed of the compressive strength test was 0.5 MPa/s, while that of the splitting strength test was 0.1 MPa/s. The three-point bending test was displacement-controlled, with a loading speed of 0.1 mm/min. The span of the three-point bending specimen was 300 mm. The test device for the above-mentioned tests is shown in Figure 2. 

#### 2.3.2. DIC Test

DIC technology is a non-destructive measurement method that can obtain the full-field strain information of a specimen surface. In DIC analysis, a reference image is divided into many small blocks (as seen in Figure 2). These blocks are extracted and compared with the previous image to obtain the information of full-field strain [24]. It is worth noting that to use the DIC technology, scattered spot preparation was required on one of the surfaces of each specimen before testing. The process of scattered spot preparation was as follows: First, the surfaces of the specimens were sprayed with a white color. Then, the specimens were stored at room temperature for 10–15 min to dry completely. After that, black spots were randomly sprayed on the surfaces of the specimens. Finally, the black spots were completely dry. Then, the specimens could be tested. During the test, a camera with a frequency of 5 Hz and a resolution of 1920 × 1080 was used to photograph the surface of a specimen with scattered spots during loading. After the test was completed, the images obtained from the camera were imported into the analysis software (VIC-2D) to process the full-field strain distribution during the loading of the specimen.

## 3. Results and Analysis

### 3.1. Failure Pattern

**Figure 3:**
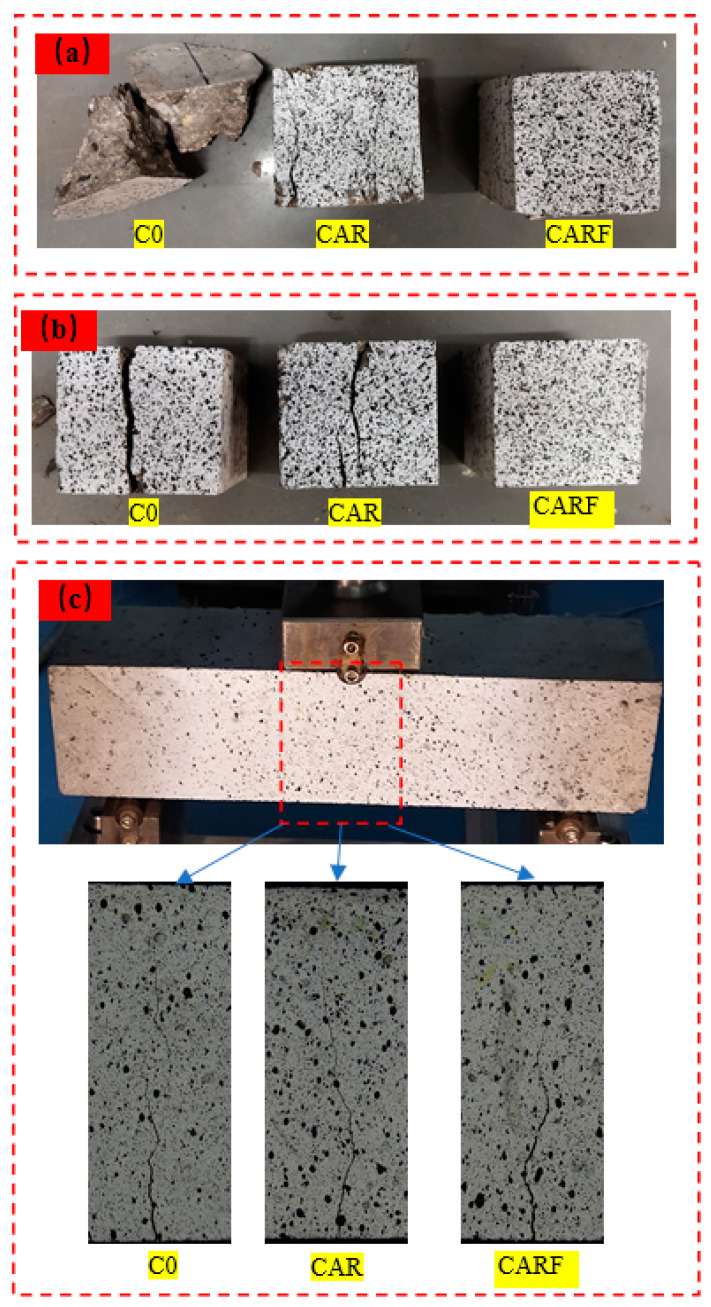
Failure patterns of specimens under (**a**) compressive, (**b**) splitting, and (**c**) three-point bending actions.

Figure 3 shows the failure patterns of the specimens under different loading modes. It can be observed in Figure 3a,b that reference specimen C0 exhibited brittle damage with a loud cracking sound under the compressive and splitting loads. In particular, the damage pattern of specimen C0 showed a typical “8” shape under the compressive load, and specimen C0 was divided into two parts under the splitting load. Moreover, although the specimen (CAR) incorporating rubber particles and polymer latex powders showed multiple macroscopic cracks on the surface, the specimen remained whole. In addition, the surface of the specimen (CARF) containing rubber particles, polymer latex powders, and polyethylene fibers had no evident macroscopic cracks, but many fine cracks could be found in the surface of the specimen. Moreover, specimen CARF maintained good integrity. As shown in Figure 3c, all specimens cracked form the middle of the bottom under the three-point bending load, and further observation revealed that the crack path of specimen C0 was the shortest, which was followed by specimens CAR and CARF. The most tortuous damage path was found in specimen CARF. Overall, the incorporation of rubber particles and polymer latex powders could improve the brittle fracture characteristics of concrete and increase the fracture path, and the further addition of polyethylene fibers could significantly inhibit the expansion of macroscopic cracks in concrete and made this fracture path the most tortuous.

### 3.2. Mechanical Properties

#### 3.2.1. Strength

**Figure 4:**
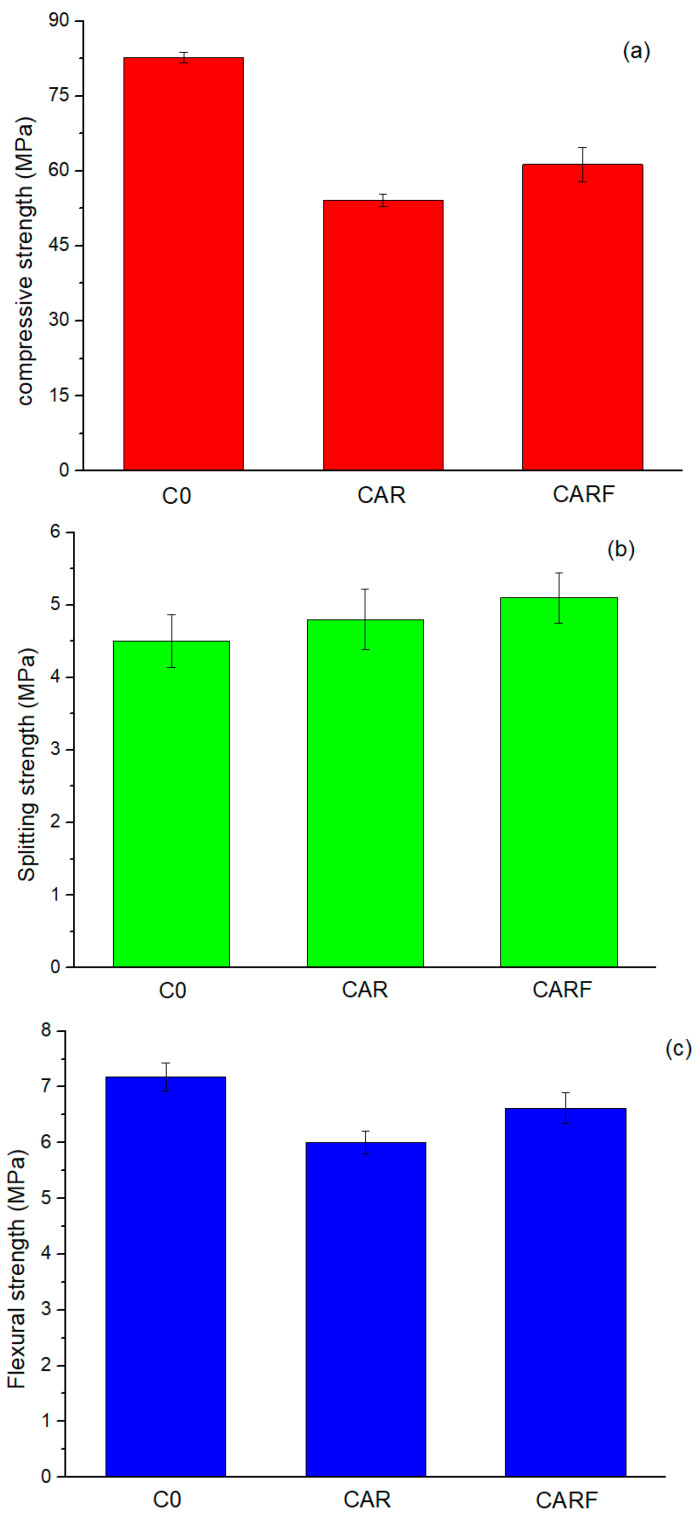
(**a**) Compressive strength, (**b**) splitting strength, and (**c**) flexural strength of specimens incorporating toughening materials.

Figure 4 shows the compressive strength, splitting strength, and flexural strength of the specimens incorporating toughening materials. It was found that the incorporation of toughening materials reduced the compressive strength and flexural strength of the specimens, but it enhanced the splitting strength of the specimens. Specifically, compared with specimen C0, the compressive and flexural strengths of specimen CAR decreased by 34.6% and 16.4%, respectively. Moreover, the splitting strength values of specimens CAR and CARF increased by 6.7% and 13.3%, respectively, compared to specimen C0. This indicates that the introduction of toughening materials could improve the splitting strength of high-strength self-compacting concrete. 

**Figure 5:**
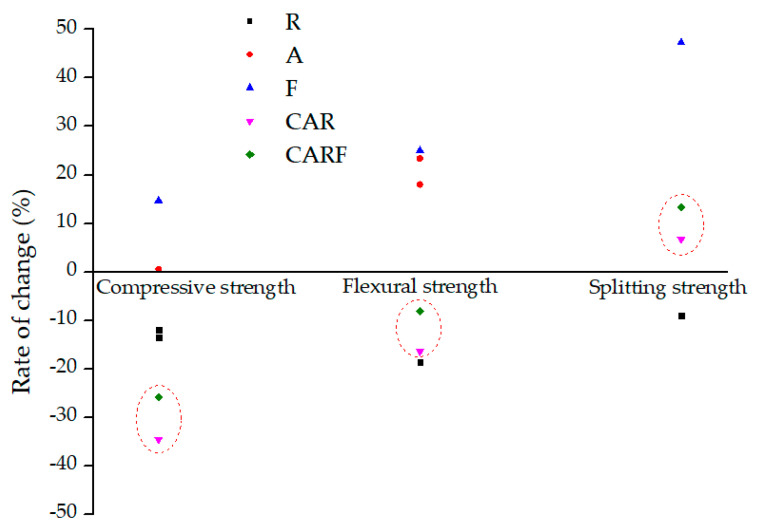
The influence rates of toughening materials on the strength of concrete.

The influence rates of toughening materials on the strength of concrete in the existing research is summarized in Figure 5. R [25,26,27], A [28,29], and F [22,30] represent the specimens containing only rubber particles, polymer latex powders, and polyethylene fibers, respectively. The dosages of the toughening materials were the same as in this study. It was found that the influence rates of compressive strength, flexural strength, and splitting strength of specimen R were lower than 0, but those of specimens A and F were higher than 0. This demonstrates that, in general, the addition of rubber particles exhibited a negative effect on the mechanical properties of cement-based materials. However, improved mechanical properties could be obtained by incorporating polymer latex powders and polyethylene fibers. Overall, the mechanical properties of concrete were at an intermediate level after adding multiple toughening materials. The toughening effect of triple-doping was better than that of double-doping, which means that the toughening effect increased with the increase in toughening material types.

#### 3.2.2. The Ratio of Flexural Strength to Compressive Strength (*f*_f_/*f*_c_)

**Figure 6:**
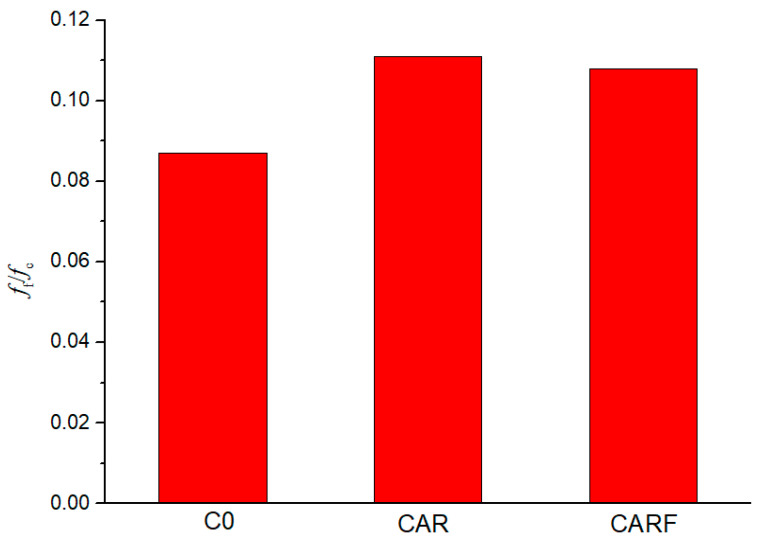
The ratios of flexural strength to compressive strength (*f*_f_/*f*_c_) of specimens incorporating toughening materials.

The ratio of flexural strength to compressive strength (*f*_f_/*f*_c_) is a common measurement for evaluating the toughness of concrete materials [6,31,32]. The higher the *f*_f_/*f*_c_, the better the toughness of the concrete. Figure 6 gives the ratios of flexural strength to compressive strength (*f*_f_/*f*_c_) of specimens incorporating toughening materials. It can be observed that the *f*_f_/*f*_c_ values of specimens CAR and CARF increased with the addition of toughening materials compared with specimen C0. In particular, the *f*_f_/*f*_c_ of specimen C0 was around 0.087, and the *f*_f_/*f*_c_ values of specimens CAR and CARF were both greater than 0.100. This suggests that the incorporation of toughening materials could enhance the toughness of high-strength self-compacting concrete.

### 3.3. Strain and Displacement Evolution

#### 3.3.1. Strain Field

**Figure 7:**
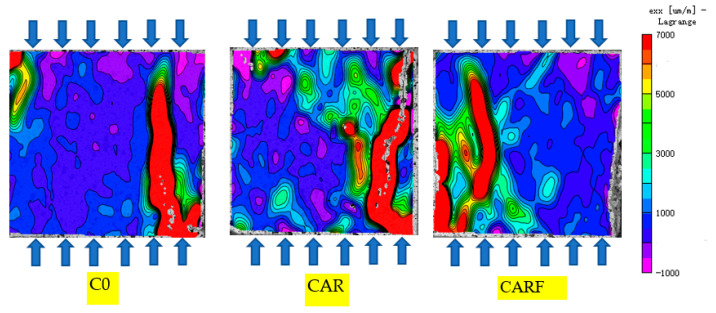
Strain fields under compressive loads of specimens incorporating toughening materials.

**Figure 8:**
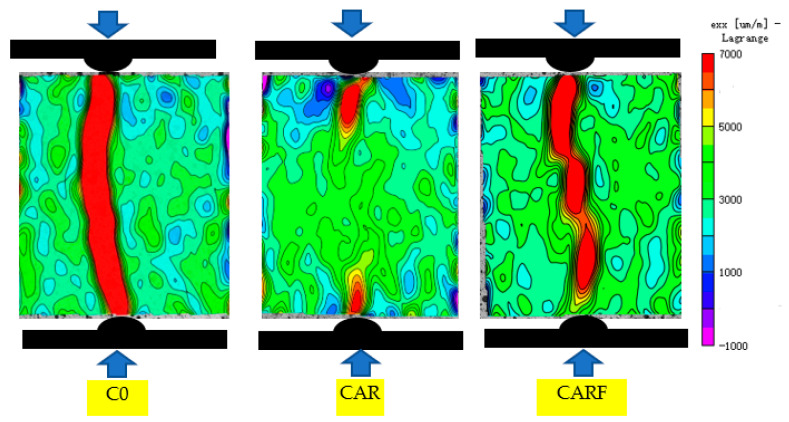
Strain fields under splitting loads of specimens incorporating toughening materials.

**Figure 9:**
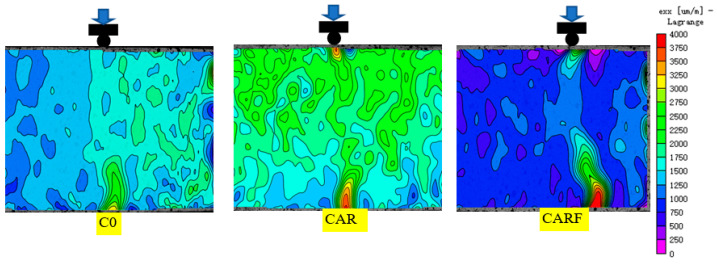
Strain fields under flexural loads of specimens incorporating toughening materials.

Strain fields under compressive, splitting, and flexural loads of specimens incorporating toughening materials are presented in Figure 7, Figure 8 and Figure 9, respectively. The distribution and development of cracks in the specimens could be clearly seen in the strain fields. As shown in Figure 7, the cracks on the surface of specimen C0 were single compared with specimens CAR and CARF, and an obvious strain concentration could be observed. Moreover, with the introduction of the toughening materials (i.e., rubber particles and polymer latex powders), the cracks in specimen CAR became tortuous and the green cloud points in the strain field increased. This implies that the strain was distributed to more areas of the specimen. Furthermore, the addition of polyethylene fibers resulted in a more tortuous crack path in specimen CARF, and more green cloud points could be observed in the strain field. It can also be seen that the main crack width of specimen CARF was narrower compared to specimen CAR. The above phenomena further illustrated that the strain distribution was the widest in specimen CARF and that the hybrid of multiple toughening materials played a role in inhibiting the development of macroscopic cracks.

As demonstrated in Figure 8, the cracks in specimen C0 developed rapidly under the splitting load, resulting in the specimen splitting into two parts. Specimen CAR exhibited the phenomenon of crack aggregation and expansion at the loading end, which indicated that the rubber particles and the polymer latex powders inside the specimen played a role in retarding crack expansion. Moreover, in specimen CARF, the cracks were gathered on the damaged surface, and the cracks were connected under the splitting load. This indicated that with the further addition of polyethylene fibers, the strain expansion path was deflected and the strain was redistributed on the damaged surface under the splitting load and that the toughening materials performed well in crack resistance.

As shown in Figure 9, it can be observed that there was one tensile crack in the middle of specimen C0, and the crack development was in the vertical direction, which was consistent with the reference of [33]. The color of the crack tip of specimen C0 was lighter than those of the specimens CAR and CARF, implying that specimen C0 had the smallest ultimate strain compared to specimens CAR and CARF. Concurrently, specimen CARF exhibited the maximum ultimate strain. This was attributed to the fact that the ability to accumulate deformation of specimen C0 was worse than those the specimens CAR and CARF, thus demonstrating that the addition of the toughening materials enhanced the ability to accumulate deformation of high-strength self-compacting concrete.

#### 3.3.2. Crack Opening Displacement (COD)

**Figure 10:**
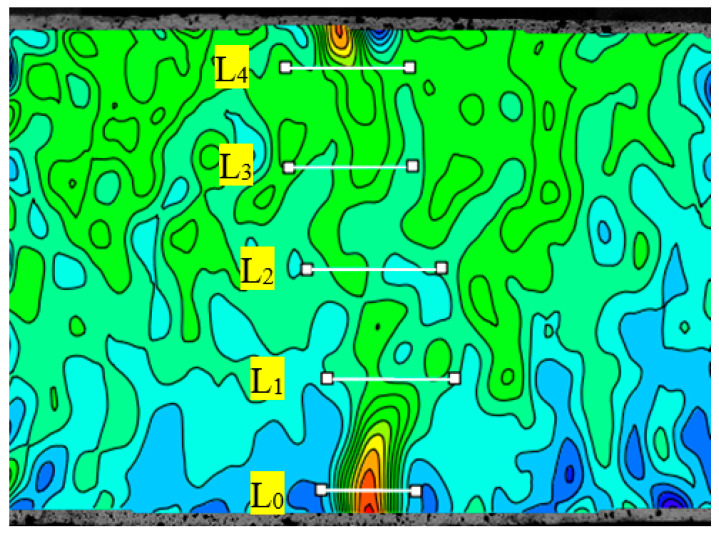
A schematic diagram of the virtual extensometers of a specimen (taking specimen CAR as an example).

**Figure 11:**
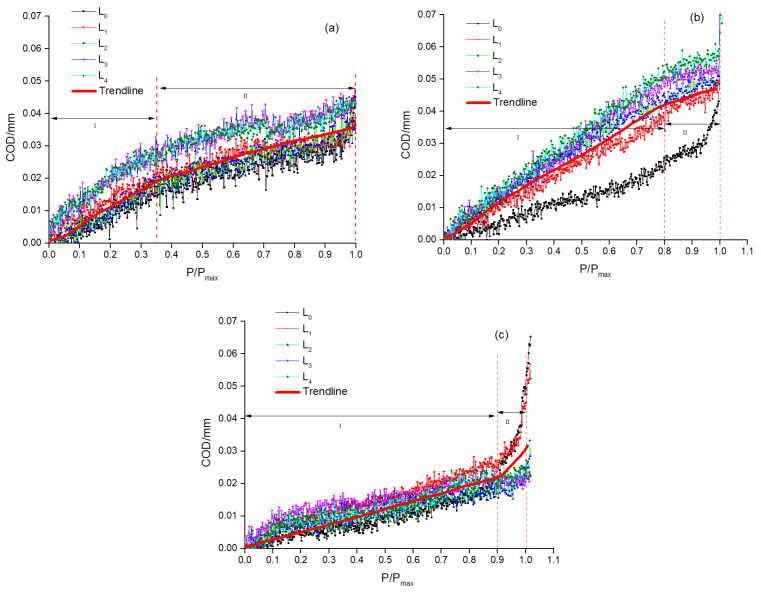
The relationships between the COD and P/P_max_ of specimens (**a**) C0, (**b**) CAR, and (**c**) CARF.

From the previous analysis, it was known that the stresses in the specimens were concentrated at their bottoms under the flexural load and that crack formation and connection led to the damage of the specimens with the increase in the load. Incorporating toughening materials brought about changes in both the crack morphology and the crack opening displacement (COD) of concrete. In order to quantitatively evaluate the influences of different toughening materials on the toughness of high-strength self-compacting concrete, we used DIC technology to analyze the displacement field under a flexural load and then obtained the influences of different toughening materials on the toughness of high-strength self-compacting concrete by quantitatively calculating COD. The specific process of calculating COD was as follows: First, the main crack was found in the strain field, and the first virtual extensometer (L_0_) was set across the main crack and at a distance of about 5 mm from the bottom of the specimen. After that, the virtual extensometer was arranged every 20 mm along the main crack to the top of the specimen, with a total of five virtual extensometers. A schematic diagram of the virtual extensometers of a specimen is presented in Figure 10. The COD could be obtained by calculating the displacement of the above virtual extensometer under different loads, and the load (P) was divided by P_max_ to carry out a normalization treatment (i.e., P/P_max_). The relationship between COD and P/P_max_ is shown in Figure 11.

As illustrated in Figure 11, the COD evolution of all specimens could be classified into two stages; specifically, the first was the linear growth stage (I) and the second was the plastic yielding stage (II). In stage I, it was found that the linear growth stage of specimen C0 was before 0.35 P_max_, and that of specimens CAR and CARF was before 0.8 P_max_ and 0.9 P_max_, respectively. This indicates that the linear growth stage of crack expansion in the concrete was elongated with the introduction of toughening materials; in other words, the toughening effect of the toughening materials (i.e., polyethylene fibers, rubber particles, and polymer latex powders) on the high-strength self-compacting concrete matrix resulted in an enhancement in the deformability of the concrete matrix. In addition, the COD of specimen C0 increased suddenly after reaching stage II, and specimen C0 failed. Although the COD of specimens CAR and CARF also increased after reaching stage II, specimens CAR and CARF still had load-bearing capacity. This demonstrates that the toughening materials provided a good energy absorption effect in high-strength self-compacting concrete, allowing the damaged energy inside the concrete to be released slowly and smoothly.

**Figure 12:**
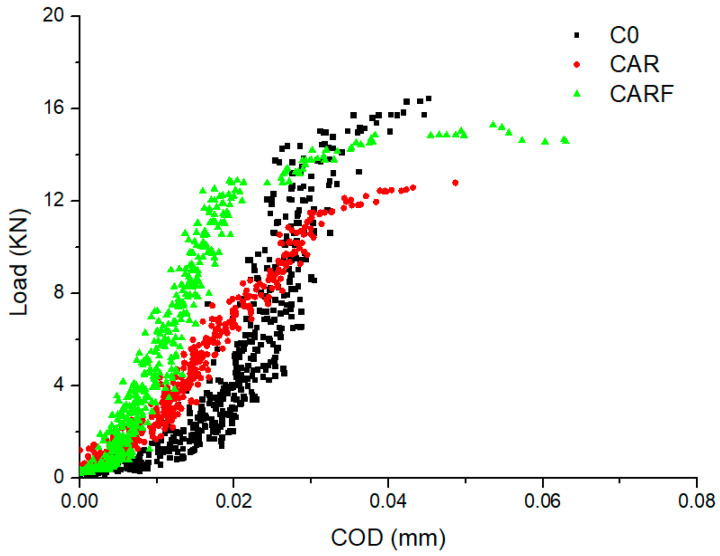
The load–COD curves of specimens with different toughening materials.

Figure 12 shows the load–COD curves of specimens with different toughening materials. It can be observed that the load–COD curves of specimens CAR and CARF were higher overall than that of specimen C0. The characteristic parameters of the specimens obtained from Figure 12 are shown in Table 3. The energy absorption capacity was the area enclosed by the load–COD curve and the x-axis [34]. It can be found in Table 3 that the COD of specimen CARF was the largest, which was followed by specimens CAR and C0. It is noteworthy that adding toughening materials reduced the peak load of the specimen to a certain extent, which was consistent with the previous research of our team [35]. Moreover, compared with specimen C0, the energy absorption capacities (S) of specimens CAR and CARF were enhanced by 30.2% and 78.5%, respectively. In particular, the energy absorption capacity of specimen CARF, which was mixed with polyethylene fibers, was higher than 0.5. This suggests that the incorporation of polyethylene fibers had the best effect on improving the toughness of the specimens compared with rubber particles and polymer latex powders. In terms of the toughening mechanism, the polymer latex powder could not only form a polymer film to improve the interface transition zone (ITZ) between the aggregate and the cement matrix but also enhanced the energy absorption capacity of hydration products at the micro-scale [36,37]. Rubber particles that had larger deformation and energy absorption could act as a “flexible skeleton” and effectively alleviate the stress concentration at the crack tip of the concrete [38,39]. Additionally, the good bridging effect of the polyethylene fibers enhanced the ability of the concrete to absorb external energy [40]. Overall, each material exhibited its advantages. The synergistic effect of three types of toughening materials (including polymer latex powders, rubber particles, and polyethylene fibers) contributed to good deformation characteristics and high toughness of high-strength self-compacting concrete.

## 4. Conclusions

Based on the results of this study, the following conclusions can be drawn:

1. DIC technology could successfully assess the crack expansion path and strain field of high-strength self-compacting concrete under an external load. The incorporation of rubber particles, polymer latex powders, and polyethylene fibers could increase the fracture path and inhibit the macrocrack expansion of high-strength self-compacting concrete. Moreover, the fracture mode of concrete with toughening materials changed from brittleness to ductility.
2. The addition of toughening materials, i.e., rubber particles, polymer latex powders, and polyethylene fibers, reduced the compressive and flexural strengths of high-strength self-compacting concrete but enhanced its splitting strength.
3. Incorporating toughening materials led to the redistribution of internal strains and the diminishment of stress concentration in high-strength self-compacting concrete under compressive and splitting loads, thus exhibiting a significant effect of inhibiting crack expansion. Meanwhile, the addition of toughening materials increased the ultimate strain under a flexural load, which improved the deformation capacity of the concrete.
4. The evolution of crack opening displacement (COD) in high-strength self-compacting concrete could be divided into two stages, including the linear growth stage and the plastic yielding stage. Adding toughening materials could extend the linear growth stage and provide a good energy absorption effect in the plastic yielding stage of high-strength self-compacting concrete.
5. The COD and energy absorption capacity of high-strength self-compacting concrete were improved by incorporating toughening materials. The best improvement was observed with the hybrid of rubber particles, polymer latex powders, and polyethylene fibers. This indicates that multiple types of toughening materials could effectively enhance the toughness of high-strength self-compacting concrete.

## Figures and Tables

**Table 1:** Chemical compositions and physical properties of binder materials.

	Mass Fraction (%)								LOI	Density (kg/m^3^)	Specific Surface Area (m^2^/kg)
	SiO_2_	Al_2_O_3_	Fe_2_O_3_	CaO	MgO	SO_3_	K_2_O	Na_2_O			
PC	20.84	3.95	3.19	59.62	3.56	3.36	0.82	0.18	1.76	3120	365
FA	40.72	20.94	5.24	5.52	1.36	1.59	1.58	1.09	2.1	2150	405
SF	96.21	0.31	1.50	1.50	0.26	1.26	0.33	0.93	3.9	2100	25,100

**Table 2:** Mix proportions and fresh properties of concrete.

Serials	Mix Proportion (kg/m^3^)										Fresh Properties	
	PC	FA	SF	W	SP	S	G	A	R	F	Extensibility (mm)	T_500_ (s)
C0	365	139.5	15.5	149.7	7.8	870.4	805	-	-	-	630	4.0
CAR	328.5	125.6	14.0	152	7.8	783.3	805	38	32	-	615	4.5
CARF	328.5	125.6	14.0	152	8.1	783.3	805	38	32	3	580	5.5

**Table 3:** Characteristic parameters of specimens.

Specimens	COD (mm)	Peak Load (KN)	Energy Absorption Capacity (S) (N·m^−1^)
C0	0.0440	16.5	29.47
CAR	0.0487	12.9	38.37
CARF	0.0535	15.3	52.61

## Data Availability

Not applicable.
